# A Clinician’s Brief Guide to the Mental Capacity Act

**DOI:** 10.1093/medlaw/fww018

**Published:** 2016-12-22

**Authors:** Laura Pritchard-Jones

**Affiliations:** School of Law, Keele University

What are the basic principles of mental capacity law in England and Wales and how do they apply in practice? What happens when an adult has a mental or physical incapacity and so is unable to manage their own affairs, yet owns property in several different jurisdictions? Or becomes incapacitated in a jurisdiction other than where they usually live? The importance of these areas is well rehearsed, and health care law academics are now well versed in reciting why these areas of law are of increasing significance. It is well known, for example, that the number of people diagnosed with dementia is expected to continue rising and it is increasingly likely that practitioners from both medicine and social work will be required to have a working knowledge of the provisions of the Mental Capacity Act 2005. Similarly, and as Paul Lagarde notes in his Foreword to *The International Protection of Adults*, there is an increasingly international dimension to mental capacity law given that socio-economic changes in the last 50 years have resulted in legal practitioners having to contend with situations where an adult may have more than one nationality, own property in different jurisdictions, or becomes incapacitated while abroad (p xi).

Both the books that form the subject of this review are concerned with these overlapping areas of law—what to do when someone becomes incapacitated and requires help or assistance managing their property or personal affairs. *A Clinician’s Brief Guide to the Mental Capacity Act* aims to provide a comprehensive overview of the 2005 Act—the statute that has regulated the treatment of those who lack mental capacity in England and Wales since 2007. The predominant focus of *The International Protection of Adults*, in contrast, is the Hague Convention on the International Protection of Adults (hereinafter Hague 35). This was an attempt to set up a mechanism with which to facilitate cross-border protection of adults with either a mental or physical disability that renders them unable to protect their own interests or assets. As Frimston and colleagues state, Hague 35:

provides a framework upon which those concerned with securing the rights of some of the most vulnerable in societies around the world can build to ensure that they do not slip between the cracks of different jurisdictions, and it provides a starting point to bring about the effective cross-border acceptance of at least some forms of anticipatory measures across borders. (para 10.08)

Each book is divided into chapters or parts that deal with specific aspects of the relevant legal areas that form the basis of the discussion. *A Clinician’s Brief Guide* begins, in Chapters 1 and 2,^1^ with a general overview of the area and background information on the legal context to the 2005 Act. Specific areas of the Act itself are then considered—an outline of the principles in relation to capacity assessment, best interests assessment, advance decisions, Independent Mental Capacity Advocates and research, the Deprivation of Liberty Safeguards, the practical aspects of the Court of Protection, and the relationship between the Mental Capacity Act and the Mental Health Act.^2^ *The International Protection of Adults* is divided into four parts and begins, in Part I,^3^ with a general overview of this area, including a synopsis of key concepts and definitions in private international law and civil or common law jurisdictional differences. Part II includes a more detailed overview of the provisions and substance of Hague 35,^4^ and Part III provides an overview of relevant capacity law in various different jurisdictions,^5^ before concluding with a number of case studies in Part IV.^6^ There are also a number of appendices, including the text of Hague 35 itself.

Both books are clearly aimed at practitioners—*A Clinician’s Brief Guide* is, as is evident from its title, geared towards providing a basic overview of the provisions of the 2005 Act for clinicians and social work practitioners, while *The International Protection of Adults* provides an in-depth manual of this area for legal practitioners. As such, neither book has a central analysis or argument. In the light of this, this review will draw out the positive and negative aspects of each book, as well as highlighting some interesting dimensions that are raised in them. In particular, it is noteworthy that each book raises issues that have, so far, generally been confined to academic debate. This highlights the fact that the debates on mental capacity law that have thus far been predominantly confined to academia must be taken seriously on a practical level.

With regards to the practical implications of largely academic debates on mental capacity law, it is frequently noted in *The International Protection of Adults* that the United Nations Conventions on the Rights of Persons with Disabilities (UNCRPD) is becoming increasingly important.^7^ Indeed, because of this Convention the law in this area in many jurisdictions is now in a state of flux. This is due to Article 12, which requires state parties to provide necessary support for persons with disabilities to exercise their legal capacity^8^ and any measures relating to the exercise of their legal capacity must respect their ‘will and preferences’.^9^ These two provisions were also stressed by the Committee on the Rights of Persons with Disabilities General Comment No 1,^10^ which goes on to say that any ‘substituted’ decision-making process, such as the best interests approach under the Mental Capacity Act for example, will never be acceptable,^11^ and in the event that a person’s will and preferences are not discernible, authorities must proceed on the basis of the ‘best interpretation of their will and preferences’.^12^ As a result of this, many states now have to rethink their mental capacity law so that they comply with the provisions of the UNCRPD and the General Comment. Yet the ideas promulgated by the UNCRPD and the General Comment have not received wholehearted approval among academic commentators. John Dawson, for example, suggests that the provisions of the UNCRPD and its General Comment are full of ‘ambiguities and inconsistencies’ and are, ultimately, unrealistic in their wholesale rejection of substituted decision-making paradigms.^13^ This is particularly so in relation to those with significant mental impairments, such as severely advanced dementia, who may not feasibly be supported to take any decision at all.

This argument is also noted in *The International Protection of Adults* (para 3.35), and thus presents a welcome scepticism of the UNCRPD’s solely supported decision-making vision for mental capacity law and adult protection. Frimston and colleagues also suggest that there is a clear distinction between ‘legal’ capacity and ‘mental’ capacity (para 1.09). This is particularly salient because Article 12 is, in fact, focused on ensuring that people with disabilities are guaranteed the same right to *legal* capacity, which does not automatically bar a finding of *mental* incapacity. This debate has, to date, manifested itself more within the realms of academic discussion as to how compliant the 2005 Act, for example, is with the UNCRPD.^14^ Their overt referencing in this book indicates that they are, in fact, debates that legal practitioners in any jurisdiction must keep at the forefront of their practice. Despite this, it is noteworthy that the impact of the UNCRPD on English and Welsh mental capacity law does not feature in *A Clinician’s Brief Guide*. This might indicate that while lawyers may be attuned to the issues that the UNCRPD raises in relation to mental capacity law, clinicians and social workers may not be and international human rights law may be far removed from their daily practices.

Frimston and colleagues also note that the drafters of Hague 35 took as their basis the Hague Conventions for the protection of children (Hague 28 and Hague 34) (paras 9.07, 10.02). This approach to adult safeguarding, drawing on similar measures for the protection of children, is familiar in other contexts, such as European Convention on Human Rights jurisprudence. In *HM v Switzerland*,^15^ for example, a case involving the alleged deprivation of liberty of a woman in her 80s as a result of neglectful and unacceptable conditions at home where she lived with her son, the European Court of Human Rights drew on the case of *Nielsen v Denmark*,^16^ involving the deprivation of liberty of a child, in deciding that HM had not been ‘deprived of her liberty’ contrary to Article 5(1) of the European Convention. Drawing analogies between adults who lack capacity and children has attracted criticism, particularly in discussions of adult protection. Writing in the US context, Nina Kohn argues that such an approach in relation to the mandatory reporting of suspected abuse, and in particular where states have adopted almost identical wording for the mandatory reporting of elder abuse as for the mandatory reporting of suspected child abuse, constitutes an infringement of the adult’s rights.^17^ Conflating or directly implementing systems that protect children on adults who lack capacity risks eliminating the adults’ rights entirely and camouflages the fact that children themselves have their rights curtailed in many different domains with a view to protecting them until they reach adulthood. Such an approach, Kohn argues, is inappropriate for adults who lack capacity and risks paternalistically hiding their voice entirely from safeguarding proceedings. Frimston and colleagues discuss the relative merits of this from a practical perspective, particularly in Chapter 7,^18^ and refer briefly to this debate:

even where an adult is considered … to lack the ability to take their own decisions, one of the most important trends over the past half-century in both common and civil law jurisdictions has been away from the model of treating them as ‘big children’ and towards seeking … to recognize and promote their autonomy. (para 7.28)

The broader comparative benefits and drawbacks of this approach, both practically and theoretically, under Hague 35 may well be a fruitful area for future research.

This recognition of academic debate can also be found, on occasions, in *A Clinician’s Brief Guide*. On page 30, Brindle and colleagues state that ‘the threshold for determining whether someone lacks capacity depends, in part, on the consequences of the decision and the resulting harm to that person’. Similarly, in *The International Protection of Adults* it is said that ‘if an individual repeatedly makes unwise decisions then a higher capacity assessment threshold applies’ (para 11.53). It is arguable that the terminology in section 1(4) of the 2005 Act itself, ‘A person is not to be treated as unable to make a decision *merely* because he makes an unwise decision’, allows for the consequences of a decision to be *one of* the features that capacity assessors take into consideration when assessing whether someone lacks capacity,^19^ but that the consequences of making a decision should never be the *sole* reason that a person is deemed to lack capacity. Although David Gibson has highlighted this tension behind the capacity assessment process under the Mental Capacity Act,^20^ it is antithetical and, arguably, contrary to the substantive provisions and ethos of the Act itself to suggest that there is a ‘higher’ capacity threshold for those who make repeatedly unwise decisions, or that the threshold depends on the consequences of the decision or the resulting harm. The assertions that the authors of both books make sit uncomfortably with the fundamentally functional nature of capacity assessments in the Act as being able to understand, retain, use or weigh information, and communicate a decision,^21^ as well as the ethos of de-stigmatisation behind section 1(4) itself. The recent House of Lords Select Committee’s post-legislative scrutiny of the 2005 Act,^22^ as well as empirical evidence,^23^ highlight that assuming someone lacks capacity because they are making an unwise decision or because they disagree with the professional opinion, frequently occurs in practice, and operates to the detriment of those who are deemed to lack capacity and functions so as to hide their ‘voices’ from the assessment process and best interests decision-making.

Despite these points of interest, neither book is without its flaws. While *A Clinician’s Brief Guide* is clearly written, particularly Chapter 7 on the notoriously complicated area of the Deprivation of Liberty Safeguards, there are a number of oversights. One is the operation and application of the offence of wilful neglect or ill treatment in section 44 of the 2005 Act. This oversight is particularly noticeable given that there is now a generalised offence of this nature,^24^ which no longer only applies to those who lack capacity or are receiving treatment for a mental disorder under the Mental Health Act.^25^ There are also some inaccuracies, albeit inaccuracies that may be attributable to simple lexical confusion. On page 3, for example, Brindle and colleagues state that ‘[a] number of matters fall outside the scope of the MCA’ and cite examples such as marriage and sexual relationships. While it *is* accurate to state that it is not possible for best interests assessors to *consent to* marriage or sexual relations in the best interests of someone who lacks capacity to do these things,^26^ it is not entirely accurate to state that these decisions are altogether outside the scope of the 2005 Act. In fact, it is still possible to be deemed to *lack capacity* under the Act to consent to sexual relations or marriage.^27^ Likewise, *The International Protection of Adults* also has its weaknesses. One of these is the amount of information covered and its breadth. This is apparent throughout the book but is particularly noticeable in Part I, which deals with the substance and elements of private international law, and Part II, which provides a full overview of all the provisions contained within and the operation of Hague 35. Because of this, readers unfamiliar with private international law as a discipline and its associated terminology may find these section dense and difficult to follow and digest, despite Frimston and colleagues’ attempts to provide definitions. They do provide case examples later in the book,^28^ but the earlier parts may have benefitted from similar examples running through them, to add clarity and demonstrate how these provisions operate.

Nevertheless, both books have much to commend them to audiences from medical, social work, and legal disciplines. *The International Protection of Adults,* for example, will be of use to anybody wanting a more in-depth analysis of a particular jurisdiction’s provisions for mental incapacity, as well as those who want detailed information about such cross-border issues as is covered by the provisions contained in Hague 35, while *A Clinician’s Brief Guide* will be useful for anyone unfamiliar with this area of law and who wants a clearly written and basic overview of the provisions of the Mental Capacity Act 2005.

## Footnotes

1 ‘The Legal Framework: The Mental Capacity Act, the Human Rights Act and Common Law’ and ‘The Mental Capacity Act and the Authority to Treat’, respectively.

2 ‘Assessment of Capacity’, ch 3; ‘Best Interests’, ch 4; ‘Alternative Authority – Planning for the Future’, ch 5; ‘Independent Mental Capacity Advocates and Regulation of Research’, ch 6; ‘Deprivation of Liberty Safeguards’, ch 7; ‘The Court of Protection, Clinically Relevant Judgments from the Courts and Writing Reports’, ch 8; ‘The Mental Health Act’, ch 9.

3 ‘Overview Chapters’.

4 ‘The Cross-Border Protection of Adults: Hague 35’.

5 ‘Existing Law in Various Jurisdictions’.

6 ‘Case Studies’.

7 See, for example, paras 3.30–3.35 and 11.34–11.37.

8 United Nations Convention on the Rights of Persons with Disabilities (UNCRPD), Article 12(3).

9 ibid, Article 12(4).

10 UNCRPD General Comment No 1 ‘Equal Recognition before the Law (Art. 12)’ (11 April 2014) CRPD/C/GC/1.

11 ibid, para 17.

12 ibid, para 21.

13 J Dawson, ‘A Realistic Approach to Assessing Mental Health Laws’ Compliance with the UNCRPD’ (2015) 40 International Journal of Law and Psychiatry 70, 70.

14 P Bartlett, ‘The United Nations Conventions on the Rights of Persons with Disabilities and Mental Health Law’ (2012) 75 Modern Law Review 752; P Gooding, ‘Navigating the “Flashing Amber Lights” of the Right to Legal Capacity in the United Nations Convention on the Rights of Persons with Disabilities: Responding to Major Concerns’ (2015) 15 Human Rights Law Review 45.

15 *HM v Switzerland* [2004] 38 EHRR 17.

16 *Nielsen v Denmark* [1989] 11 EHRR 175.

17 N Kohn, ‘Outliving Civil Rights’ (2009) 86 Washington University Law Review 1053.

18 ‘Hague 35: Introduction and Background’.

19 P Skowron, ‘Evidence and Causation in Mental Capacity Assessments: *PC v City of York Council* [2013] EWCA Civ 478’ (2014) 22 Medical Law Review 631, 634.

20 D Gibson, ‘Conceptual and Ethical Problems in the Mental Capacity Act 2005: An Interrogation of the Assessment Process’ (2015) 4 Laws 229.

21 Mental Capacity Act 2005, s 3(1)(a)–(d).

22 House of Lords Select Committee on the Mental Capacity Act 2005, *Mental Capacity Act 2005: Post-Legislative Scrutiny* (The Stationery Office 2014).

23 C Emmett and others, ‘Homeward Bound or Bound for a Home? Assessing the Capacity of Dementia Patients to Make Decisions about Hospital Discharge: Comparing Practice with Legal Standards’ (2013) 36 International Journal of Law and Psychiatry 73; V Williams and others, *Making Best Interests Decisions: People and Processes* (Mental Health Foundation 2012).

24 Criminal Justice and Courts Act 2015, ss 20–21.

25 Mental Health Act 1983, s 127.

26 Mental Capacity Act 2005, s 27.

27 Although, admittedly, following *IM v LM* [2014] EWCA Civ 37, the threshold for being able to consent to sexual relations is relatively low.

28 Part IV.

